# DRBP-EDP: classification of DNA-binding proteins and RNA-binding proteins using ESM-2 and dual-path neural network

**DOI:** 10.1093/nargab/lqaf058

**Published:** 2025-05-19

**Authors:** Qiang Mu, Guoping Yu, Guomin Zhou, Yubing He, Jianhua Zhang

**Affiliations:** Agricultural Information Institute, Chinese Academy of Agricultural Sciences, Beijing 100081, China; National Nanfan Research Institute (Sanya), Chinese Academy of Agricultural Sciences/Hainan Seed Industry Laboratory, Sanya 572024, China; National Nanfan Research Institute (Sanya), Chinese Academy of Agricultural Sciences/Hainan Seed Industry Laboratory, Sanya 572024, China; China National Rice Research Institute, Chinese Academy of Agricultural Sciences, Hangzhou 310006, China; Agricultural Information Institute, Chinese Academy of Agricultural Sciences, Beijing 100081, China; National Nanfan Research Institute (Sanya), Chinese Academy of Agricultural Sciences/Hainan Seed Industry Laboratory, Sanya 572024, China; Nanjing Institute of Agricultural Mechanization, Ministry of Agriculture and Rural Affairs, Nanjing 210014, China; National Nanfan Research Institute (Sanya), Chinese Academy of Agricultural Sciences/Hainan Seed Industry Laboratory, Sanya 572024, China; State Key Laboratory of Crop Gene Resources and Breeding, Institute of Crop Sciences (ICS), Chinese Academy of Agricultural Sciences, Beijing 100081, China; Key Laboratory of Gene Editing Technologies (Hainan), Ministry of Agricultural and Rural Affairs, Sanya 572024, China; Agricultural Information Institute, Chinese Academy of Agricultural Sciences, Beijing 100081, China; National Nanfan Research Institute (Sanya), Chinese Academy of Agricultural Sciences/Hainan Seed Industry Laboratory, Sanya 572024, China

## Abstract

Regulation of DNA or RNA at the transcriptional, post-transcriptional, and translational levels are key steps in the central dogma of molecular biology. DNA-binding proteins (DBPs) and RNA-binding proteins (RBPs) play pivotal roles in the precise regulation of gene expression in these steps. Both of these two classes of proteins are nucleic acid-binding proteins (NABPs), so they exhibit significant similarity in both sequence and structure. However, traditional methods for identifying NABPs are typically time-consuming, costly, and challenging to scale up. Utilizing deep learning to classify proteins intelligently has emerged as a more efficient solution for these issues. In this study, we propose a phased classification method integrating ESM-2 with a dual-path neural network, called DRBP-EDP. Additionally, a refined approach to dataset construction is designed, resulting in the creation of high-quality protein classification datasets. The results demonstrated that the model achieved strong performance, with 90.03% accuracy in the first stage for classifying NABPs and non-nucleic acid-binding proteins, and 89.56% accuracy in the second stage for classifying DBPs and RBPs. To enhance accessibility and usability, DRBP-EDP has been developed in both executable and web-based versions, which are publicly available at https://doi.org/10.5281/zenodo.14092184 and https://github.com/MuQiang-MQ/DRBP-EDP.

## Graphical Abstract

**Graphical Abstract:**
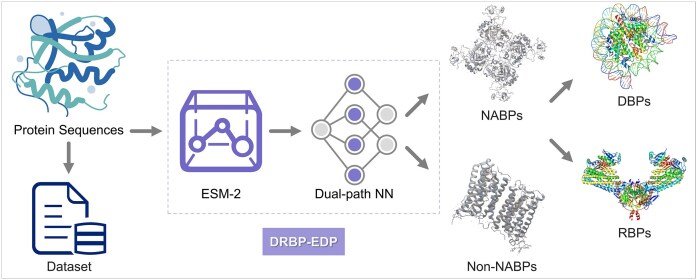

## Introduction

With the rapid advancement of next-generation biotechnologies such as gene editing, transgenics, and molecular markers, the role of proteins in the fields like agriculture and medicine has become increasingly prominent. In agriculture, DNA-binding proteins (DBPs) enhance crop resilience and disease resistance by regulating the expression of particular genes. For instance, specific transcription factors activate drought-resistant genes to increase crop survival under harsh environments [1]. Similarly, RNA-binding proteins (RBPs) are crucial in gene expression regulation [2], stabilizing mRNA and promoting its translation, enabling crops to utilize genetic resources more efficiently, thus optimizing growth and increasing yields. In medicine, DBPs are essential for gene therapies, such as correcting mutations in genetic disorders like sickle cell anemia [3], while RBPs contribute to cancer treatment by stabilizing mRNA involved in tumor suppression, enabling more targeted therapies [4]. DBPs and RBPs share considerable similarity in sequence and structure, and classifying them avoids unnecessary complexity and confusion, aids in the understanding of their specific functions and their roles in different biological processes, and facilitates the development of research tools and experimental techniques that are specifically targeted to these proteins.

Traditional methods for identifying nucleic acid-binding proteins (NABPs) primarily rely on experimental techniques such as electrophoretic mobility shift assay [5], chromatin immunoprecipitation [6], and RNA immunoprecipitation [7]. While these methods are precise, they are typically time-consuming, costly, and challenging to scale up. In addition, computational methods [8] are categorized into machine learning-based methods and structure template-based methods. The machine learning-based methods integrate feature extraction techniques (such as position-specific scoring matrix and position-specific frequency matrix) with diverse classification algorithms (including support vector machine (SVM), Random Forest (RF), extremely randomized trees (ERT), and Light Gradient Boosting Machine (LightGBM)) to develop classifiers tailored for different requirements. Representative examples include DNAbinder [9], PlDBPred [10], DPP-PseAAC [11], ProkDBP [12], DBPMod [13], StackDPPRed [14], RBPPred [15], catRAPID signature [16], RBPLight [17], iDRBP-EL [18], IDRBP-PPCT [19], and the model proposed by Varghese *et al.* [20]. In contrast, structure template-based methods leverage three-dimensional (3D) protein structural information to construct template libraries, followed by structural similarity queries between target proteins and templates to predict binding propensity. Representative examples include DBD-Hunter [21], DBD-Threader [22], SPOT [23], SPOT-Seq-RNA [24], Spalign [25], SPOT-struc [26, 27], and other structural motif-based approaches [28]. Additionally, several studies integrate sequence or structural features to predict specific binding residues, thereby determining protein binding types [29–33]. Although tools like AlphaFold [34] can predict protein structures, high-quality experimental structural data remain relatively scarce. Furthermore, the computational complexity of processing 3D structural data is substantial, and high-performance computing resources are required to train deep learning models. Therefore, utilizing deep learning [35] for sequence-based classification of DBPs and RBPs has emerged as a more efficient and feasible solution.

In the field of protein classification research, both domestic and international researchers have developed a variety of deep learning models. For example, DeepFam [36] and ProtCNN [37] utilized neural networks to process protein sequences for non-hierarchical classification of protein families. Another model, UDSMProt [38], was pre-trained on unlabeled protein sequences through self-supervised language modeling and then fine-tuned on specific tasks, demonstrating effectiveness in enzyme class prediction, gene ontology prediction, and remote homology and fold detection. Concurrently, classification models for DBPs or RBPs have also achieved significant progress. For DBPs prediction, Zhong *et al.* [39] developed a deep learning framework based on Transformer-BiLSTM architecture. Li *et al.* [40] proposed PDBP-Fusion, a hybrid CNN-BiLSTM model, while Chauhan *et al.* [41] introduced a CNN-based approach incorporating a novel feature representation strategy. In RBPs prediction, Li *et al.* [42] proposed a deep learning model that autonomously learns sequence features from a dataset comprising hundreds of thousands of sequences. Pradhan *et al.* [43] developed RBProkCNN, integrating evolutionary features with CNN, and Zheng *et al.* [44] designed Deep-RBPPred by combining RBPPred-derived protein features with CNN. Due to the substantial similarity in sequence and structure between DBPs and RBPs, binary classification of them is more complicated compared to predicting one of the proteins individually, and relevant deep learning models are relatively rare. Recent advances include hybrid architectures such as DeepDRBP-2L [45] (integrating CNN-BiLSTM), iDRBP_MMC [46] (combining CNN with motifs), PredDRBP-MLP [47] (multilayer perceptron-based framework), and DRBPPred-GAT [48] (graph multi-head attention network). Furthermore, Wu *et al.* [49] enhanced predictive accuracy through a CNN-LSTM framework, while Du *et al.* [50] developed a multiLSTM network to decode latent inter-label correlations. Hybrid approaches combining traditional machine learning with deep learning architectures have also been developed, exemplified by tools such as iDRBP-ECHF [51] and iDRPro-SC [52]. Notably, protein language models (PLMs) have been successfully applied in this domain [53, 54], demonstrating superior predictive capabilities compared to conventional methods. Collectively, these advancements provide methodologically valuable references of our study.

Despite significant advancements in previous studies, the protein classification task still encounters numerous challenges. First, the prediction accuracy of current models requires further improvement, especially when dealing with imbalanced datasets and noisy data, where their performance tends to fall short. Second, the extraction capability of protein sequence features is limited, and existing models struggle to comprehensively capture the complex features within the sequences. Moreover, the quality and quantity of data is also an essential issue. Although the amount of protein sequence is gradually increasing, high-quality annotated data remain relatively scant.

To address these challenges, this study proposes a phased classification method integrating a PLM with a dual-path deep learning network. Specifically, in the first stage, protein sequences are classified as either NABPs or non-NABPs; in the second stage, NABPs are further classified as either DBPs or RBPs. To accomplish this objective, we employ Evolutionary Scale Modeling-2 (ESM-2) with robust feature extraction capabilities and combine various deep learning techniques including Transformer, CNN, BiLSTM, and Attention to design a dual-path protein classifier named DRBP-EDP. Additionally, a high-quality protein classification dataset is created in this study to ensure the reliability of model training and evaluation. With this approach, we aim to improve the accuracy and stability of the classification model and provide an innovative tool for the investigation of protein function, thereby driving advancements in life sciences.

## Material and methods

### Datasets

**Figure 1.**
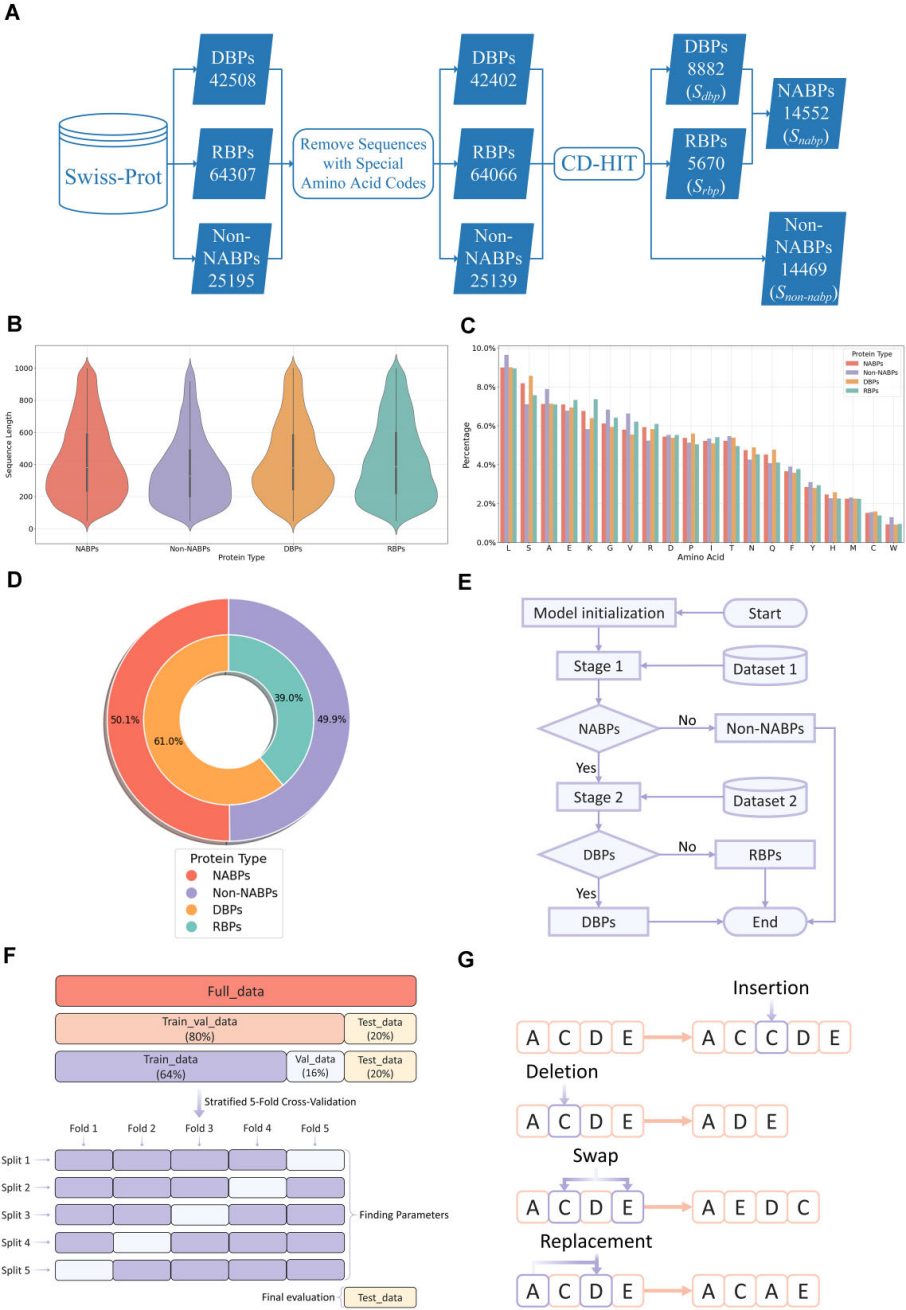
The generalized description of the data. (**A**) Data collection process. This figure describes the main steps of data screening and organization. ${{S}_{nabp}} = {{S}_{dbp}}\cup\ {{S}_{rbp}}$. (**B**) Comparison of sequence length distribution. (**C**) Comparison of amino acid composition. (**D**) Proportion of samples across different classification stages. (**E**) The main flow of model training. $Dataset{\mathrm{\ }}1 = {{S}_{nabp}}\cup{{S}_{non - nabp}}$ and $Dataset{\mathrm{\ }}2 = {{S}_{dbp}}\cup{{S}_{rbp}}$. (**F**) Data partitioning. The dataset is divided into training set (Train_data), validation set (Val_data), and test set (Test_data) by combining stratified five-fold cross-validation. (**G**) Data augmentation. There are four methods: Insertion, Deletion, Swap, and Replacement.

**Figure 2.**
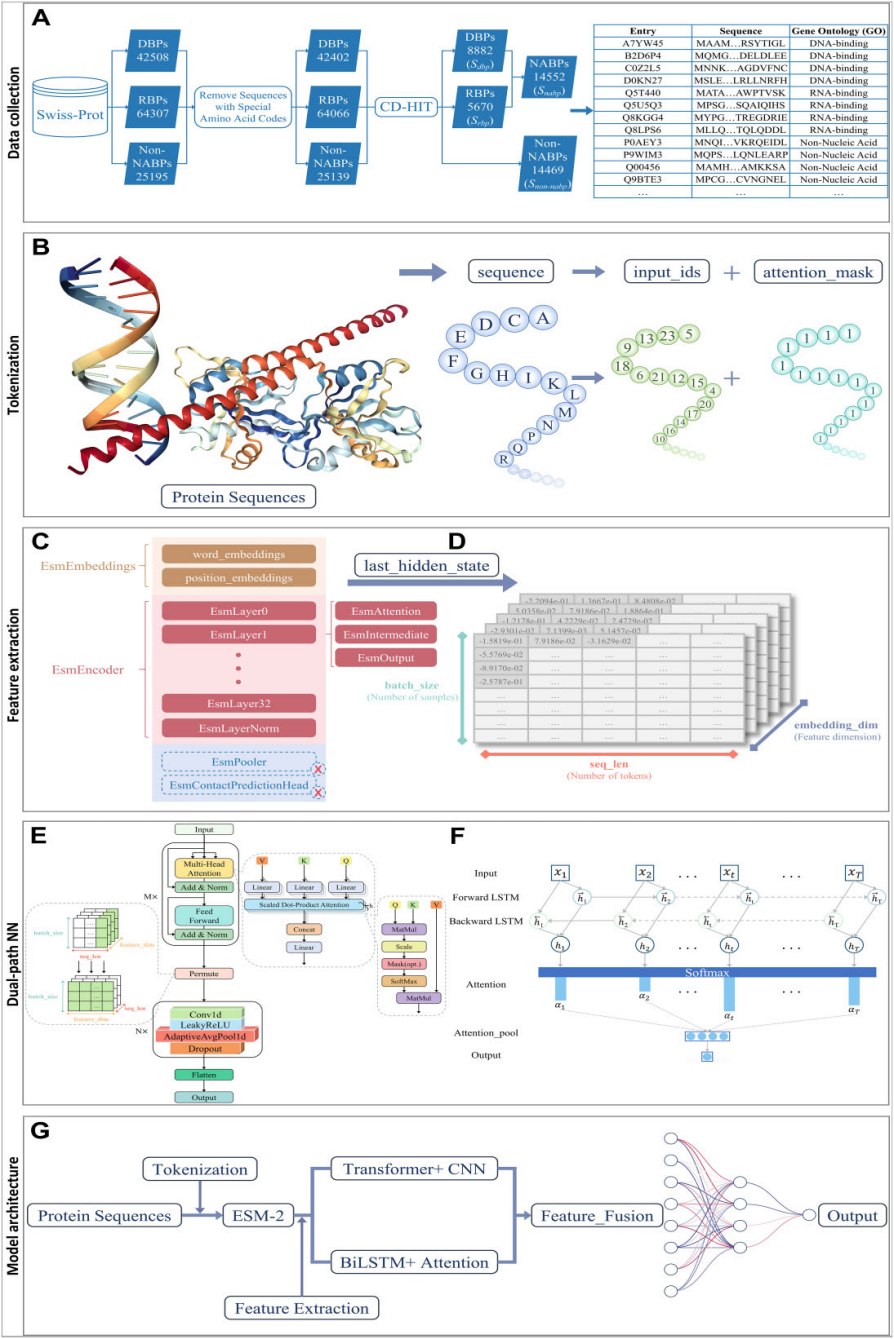
The framework of the proposed method. (**A**) Data collection and tabulation. The input file is obtained by filtering and organizing the data. (**B**) Tokenization. The sequence is converted into input_ids and attention_mask. (**C**) The fine-tuned ESM-2. (**D**) last_hidden_state, with shape [batch_size, seq_len, embedding_dim]. (**E**) Transformer-CNN. (**F**) BiLSTM-Attention. (**G**) Model architecture. Covering the entire process from input to output.

The protein dataset used in this study is sourced from Swiss-Prot [55] within the UniProt Knowledgebase (UniProtKB) of the Universal Protein Resource (UniProt) [56]. In the process of data screening and organization, we drew on the methods of dataset construction from existing studies [57–59]. The specific steps are shown in Figs 1A and 2A. We clustered protein sequences deposited in the database prior to 15 March 2024, using a minimum similarity threshold of 0.4 [60], yielding the benchmark dataset NR40. This dataset comprises 8882 DBPs, 5670 RBPs, and 14 469 non-NABPs, referred to as *S_dbp_*, *S_rbp_*, and *S_n on-na bp_*, respectively. Additional datasets (NR30, NR50, NR70, and NR90) were generated by clustering sequences at similarity thresholds of 0.3 [61], 0.5, 0.7, and 0.9. To further evaluate model generalizability, we compiled TEST236, a temporal validation set containing sequences added to the database between 15 March 2024 and 12 February 2025 using identical criteria. Furthermore, five organism-specific datasets (*A. thaliana*, *Human*, *S. cerevisiae*, *Mouse*, and *C. elegans*) were extracted from the Stage 2 independent test set of NR40 for cross-species validation. And detailed dataset statistics are summarized in Table 1. For NR40, Fig. 1B statistically profiles protein sequence lengths across categories, quantifying mean, dispersion, and skewness; Fig. 1C depicts the relative percentages of different amino acids in various types of proteins and analyses the relative abundance of each amino acid across different categories of proteins.

**Table 1.** Datasets

Dataset	Number of DBPs	Number of RBPs	Number of non-NABPs	Total number
NR30	6898	3861	12 181	22 940
NR40	8882	5670	14 469	29 021
NR50	11 799	8847	16 815	37 461
NR70	17 790	18 596	20 182	56 568
NR90	25 205	32 607	22 748	80 560
TEST236	83	77	76	236
*A. thaliana*	189	128	100	417
*Human*	126	112	177	415
*S. cerevisiae*	67	58	194	319
*Mouse*	75	21	111	207
*C. elegans*	74	20	16	110

In the first stage, we used *S_na bp_* as positive samples and *S_n on-na bp_* as negative samples to distinguish NABPs from non-NABPs, and the outer ring of Fig. 1D demonstrates the proportion of their quantities; in the second stage, we employed *S_r bp_* as positive samples and *S_d bp_* as negative samples to further categorize NABPs into DBPs or RBPs, and the inner ring of Fig. 1D depicts the ratio of their numbers. The main training process of the model is illustrated in Fig. 1E.

### Data partitioning

In this study, stratified five-fold cross-validation [62, 63] was applied in all training phases. It not only assesses the actual performance of the model more accurately but also ensures that the proportion of each category in each fold is consistent with that of the original dataset, effectively addressing the issue of class imbalance within the dataset. In Fig. 1F, to ensure the final evaluation of the model on completely independent data, we divided the full dataset into a training-validation set (Train_val_data) and an independent test set (Test_data), namely train_val_data_stage1 and test_data_stage1 in the first stage, and train_val_data_stage2 and test_data_stage2 in the second stage, avoiding data leakage [64] and model overfitting [65]. In each split, we reinitialized the model to ensure that each training session began with identical initial conditions, thus preventing interference between model states and guaranteeing fairness and consistency in the evaluation. Moreover, during the first stage of training, we saved the model state in each training evaluation. In the second stage of training, we loaded the best model state from the first stage to continue training and evaluation.

### Data augmentation

To improve the generalization and robustness of the model, we designed a systematic data augmentation strategy (Fig. 1G), introducing protein sequence variants through multiple methods:

- Randomly selecting an amino acid from the sequence and inserting it at a random position;
- Randomly selecting an amino acid and deleting it from the sequence;
- Randomly selecting two amino acids and swapping their positions;
- Randomly selecting an amino acid and replacing it with another randomly chosen amino acid.

Furthermore, we implemented a dynamic adjustment of the data augmentation probability to ensure that the data augmentation strategy works appropriately at different stages of model training. During the early phase of training, a higher data augmentation probability (e.g. 0.5) was applied to rapidly introduce data diversity, thereby mitigating the model's prior bias towards the initial data distribution. As training progresses, this probability was gradually reduced (by 10% per epoch), allowing the model to increasingly focus on the details and intrinsic patterns of the real data in the later stages, which contributes to the stable convergence and accuracy of the model.

### Overall architecture

Compared with traditional feature extraction techniques, PLMs can automatically learn high-dimensional and complex features from sequences. And the dual-path neural network overcomes the limitations of single-path architectures by processing distinct feature channels in parallel.

In this study, a model architecture (Fig. 2G) that integrates ESM-2 with a dual-path neural network is designed. Initially, protein sequences are tokenized using AutoTokenizer [66] to convert them into an input format that can be processed by the model (Fig. 2B). Specifically, each amino acid in the protein sequence is represented by a unique numerical identifier derived from the vocabulary index of the pre-trained model (ESM-2), and these identifiers collectively form the “input_ids.” Additionally, “attention_mask” is a binary matrix used to indicate which tokens the model should focus on during processing of the input, ensuring that the model only pays attention to the actual input tokens and ignores the padding tokens by masking out the padding section (padding section is marked as 0 and the actual input part is marked as 1).

After receiving the input data, ESM-2 transforms it through multiple layers to output “last_hidden_state” (with a shape of [batch_size, seq_len, embedding_dim], as shown in Fig. 2D), which contains the semantic information and contextual dependencies of each token within the sequence. Subsequently, these high-dimensional feature representations are further refined through two distinct pathways (Transformer-CNN and BiLSTM-Attention) to extract higher-level features. These advanced features are then fused through a fully connected layer to form more compact and information-rich feature representations. Finally, the fused feature vectors are passed to the classification layer to generate classification results.

### ESM-2

ESM-2 is a PLM based on the Transformer architecture [67, 68], which captures intricate relational and semantic information within sequences through unsupervised learning on tens of millions of protein sequences from the UniRef database. Compared to its predecessor, ESM-2 incorporates relative position embeddings, allowing it to handle sequences of arbitrary lengths, and integrates Rotary Position Embeddings (RoPE) [69], enabling the model to extrapolate outside the context window of its training. ESM-2 is available in several versions with varying parameter sizes, and we selected a model with 650 M parameters, whose encoder comprises multi-layer self-attention mechanisms and feed-forward neural networks for extracting feature dependencies within the sequences.

In this study, we chose not to involve the EsmPooler layer and the EsmContactPredictionHead layer (Fig. 2C) but instead used the output of the last layer of the encoder (last_hidden_state) directly for fine-tuning and froze the EsmEmbeddings layer and the EsmEncoder layer during model training to keep the pre-trained weights of these layers unchanged. In these ways, we can take full advantage of the rich features learned by the pre-trained model on large-scale protein sequences to improve performance on classification tasks.

### Transformer-CNN

In Path 1 (Fig. 2E), the Transformer encoder utilizes a multi-head self-attention mechanism to capture global dependencies, while the CNN module further extracts local features and spatial information, and the combination of the two effectively enhances the model performance. Specifically, the Transformer encoder is composed of a stack of M identical layers, each of which mainly contains two sub-layers: a multi-head self-attention mechanism and a position-wise feed-forward network [70], and each sub-layer is followed by a residual connection [71] and layer normalization [72]. That is, the output of each sub-layer is $LayerNorm( {x + subblayer( x )} )$, where $x$ is the input feature of the current layer and $subblayer( x )$ is the output of the current layer.

The scaled dot-product attention is used to analyze the similarity and correlation between amino acids in the input sequence. It obtains attention scores by computing the dot product of the query with all keys, and then uses these scores to generate a new embedding representation by performing a weighted summation on the values. The calculation is as follows:


(1) \begin{eqnarray*}
Attention\left( {Q,K,V} \right) = softmax\left( {\frac{{Q{{K}^T}}}{{\sqrt {{{d}_k}} }}} \right)V
\end{eqnarray*}

Where $Q$, $K$, and $V$ represent the matrices of queries, keys, and values, respectively, with ${{d}_k}$ being the dimensionality of the keys. In multi-head attention, the queries, keys and values are each linearly transformed into ${\mathrm{h}}$ different subspaces, where each subspace has a dimensionality of $\frac{{{{d}_{model}}}}{h}$, with ${{d}_{model}}$ representing the total dimensionality of the input. In each separate subspace, the scaled dot-product attention is applied in parallel, producing outputs from ${\mathrm{h}}$ different attention heads, with each head computed as follows:


(2) \begin{eqnarray*}
hea{{d}_i} = Attention\left( {QW_i^Q,KW_i^K,VW_i^V} \right)
\end{eqnarray*}

Here, $W_i^Q$, $W_i^K$ and $W_i^V$ are distinct parameter matrices used to project $Q$, $K$, and $V$ to the $i$th subspace. Then the outputs of all the heads are concatenated together to form a new matrix: $Concat( {hea{{d}_1},\ldots,hea{{d}_h}} )$. The spliced output, with a dimension of ${{d}_{model}}$, is subsequently linearly transformed using a matrix ${{W}^O}$ to generate the final output representation:


(3) \begin{eqnarray*}
MultiHead({Q,K,V}) = Concat({\mathit{head}}_{1},\ldots,{\mathit{head}}_{h}){W}^{O}\nonumber\\
\end{eqnarray*}

The matrix ${{W}^O}$, which is part of the model parameters, is optimized through the backpropagation algorithm during the training process. It linearly transforms the concatenated multi-head self-attention output back to the original dimensionality ${{d}_{model}}$ to further merge the information from the different heads and improve the expressive capacity of the model. This process ensures that the final output representation maintains the same dimensions as the input, ensuring the consistency and effectiveness of the model when processing in subsequent layers.

Before passing the output of the Transformer encoder to the CNN module, it needs to be dimensionally adjusted to meet the input requirements of the CNN. Specifically, the operation named “Permute” is used to rearrange the dimensionality of the tensor [73], changing its shape from [batch_size, seq_len, feature_dim] to [batch_size, feature_dim, seq_len].

The CNN module consists of *N* identical convolutional blocks. In each block, the Conv1d layer is used to extract local features from the input data [74], and its output length is calculated as follows:


(4) \begin{eqnarray*}
output\_length &=& \left( {input\_length + 2 \times padding - kernel\_size} \right)\nonumber\\ &&/stride + 1
\end{eqnarray*}

Where $input\_length$ is the length of the input features, $padding$ denotes the number of zero elements padded at the edge of the input features, $kernel\_size$ represents the size of the convolution kernel, and $stride$ refers to the step size by which the convolutional kernel traverses the input; the LeakyReLU layer introduces nonlinearity by outputting a small linear value when the input is less than zero, thereby avoiding the dying ReLU problem [75], where neurons output zero consistently; the AdaptiveAvgPool1d layer sizes the feature map to a specified output scale by applying average pooling over local regions, thereby aggregating features and reducing computational complexity; the Dropout layer randomly deactivates neurons to prevent overfitting [76], enhancing the generalization capability of the model. After the CNN module processes the data, its output is flattened into a one-dimensional vector to facilitate concatenation with features from other paths.

### BiLSTM-attention

In Path 2 (Fig. 2F), the BiLSTM captures bidirectional dependencies within the input sequence, while the Attention mechanism further extracts crucial information from the sequence. Specifically, let the high-dimensional features output by ESM-2 be denoted as ${\mathrm{x}} = [ {{{x}_1},{{x}_2},\ldots,{{x}_t},\ldots,{{x}_T}} ]$, where $t$ represents the current time step or position, $T$ refers to the length of the whole sequence (i.e. the total number of time steps), ${{x}_t}$ is the feature representation of ESM-2 for the $t$th position in the protein sequence. The BiLSTM [77, 78] computes the hidden states through the forward LSTM layer and the backward LSTM layer, respectively. First, the forward LSTM layer processes the sequence $[ {{{x}_1},{{x}_2},\ldots,{{x}_t},\ldots,{{x}_T}} ]$, computing the forward hidden state $\overrightarrow {{{h}_t}}$ at each time step sequentially from $t = 1$ to $t = T$:


(5) \begin{eqnarray*}
\overrightarrow {{{h}_t}} = LST{{M}_{forward}}\left( {{{x}_t},\overrightarrow {{{h}_{t - 1}}} } \right)
\end{eqnarray*}

Then, the backward LSTM layer processes the sequence $[ {{{x}_T},{{x}_{T - 1}},\ldots,{{x}_t},\ldots,{{x}_1}} ]$, computing the backward hidden state $\overleftarrow {{{h}_t}}$ at each time step sequentially from $t = T$ to $t = 1$:


(6) \begin{eqnarray*}
\overleftarrow {{{h}_t}} = LST{{M}_{backward}}\left( {{{x}_t},\overleftarrow {{{h}_{t + 1}}} } \right)
\end{eqnarray*}

Here, $\overrightarrow {{{h}_t}}$ is the hidden state of the forward LSTM at time step $t$, and $\overrightarrow {{{h}_{t - 1}}}$ is the hidden state of the forward LSTM at time step $t - 1$. Similarly, $\overleftarrow {{{h}_t}}$ is the hidden state of the backward LSTM at time step $t$, and $\overleftarrow {{{h}_{t + 1}}}$ is the hidden state of the backward LSTM at time step $t + 1$. These are then concatenated to form the bidirectional output representation, i.e. ${{h}_t} = [ {\overrightarrow {{{h}_t}} ,\overleftarrow {{{h}_t}} } ]$.

The Attention mechanism [79] calculates the weights for each position in the input data. The attention weight is computed using the following formula:


(7) \begin{eqnarray*}
{{\alpha }_{ij}} = \frac{{exp\left( {{{q}_i} \cdot {{k}_j}} \right)}}{{\mathop \sum \nolimits_{j = 1}^T exp\left( {{{q}_i} \cdot {{k}_j}} \right)}}
\end{eqnarray*}

Here, $i$ and $j$ are the indices of the time steps, ranging from $1$ to $T$. ${{q}_i}$ is the $i$th element of the query vector $Q$, corresponding to the $i$th time step. ${{k}_j}$ is the $j$th element of the key vector $K$, corresponding to the $j$th time step. The attention weights are then normalized using the softmax function, and a new representation is obtained by taking a weighted sum of the value vector $V$:


(8) \begin{eqnarray*}
Attention\left( {Q,K,V} \right) = \mathop \sum \limits_{j = 1}^T {{\alpha }_{ij}}{{v}_j}
\end{eqnarray*}

Where ${{v}_j}$ is the $j$th element of the $V$, corresponding to the $j$th time step. Finally, a fixed-size feature representation is obtained through average pooling and a fully connected layer.

### Hyperparameter configuration

In this study, we utilized the Optuna framework [80] to optimize the hyperparameters of our model. Ultimately, through iterative adjustments based on cross-validation accuracy and personal experience, the best hyperparameter combination was determined as follows (Table 2):

**Table 2.** The setting of hyperparameters

Hyperparameters	Value
Epochs	50
Batch_size	8
Augmentation_prob	0.5
Early_stopping_patience	4
Optimizer	AdamW
Learning_rate (pretrained, custom)	5e-6, 5e-5
Weight_decay (pretrained, custom)	1e-2, 3e-1
Scheduler (factor, patience, min_lr)	ReduceLROnPlateau (0.6, 2, 1e-6)

To maximize the advantages of the pre-trained model, we employed a lower learning rate and appropriate weight decay (L2 regularization) [81] for precise fine-tuning. Specifically, the weight decay was set to zero for parameters containing “bias” and “LayerNorm.weight” in the pre-trained model to avoid unnecessary regularization. The custom dual-path network structure was trained with a higher learning rate and weight decay to balance the fine-tuning of the pre-trained model and the need to learn new features, thereby mitigating conflicts and instability during the training process. Furthermore, a learning rate scheduler was implemented to automatically adjust the learning rate based on validation loss, while an early stopping mechanism [82] was combined to prevent overfitting and optimize computational efficiency.

### Loss function

Binary Cross-Entropy with Logits Loss (BCEWithLogitsLoss) [73] is a loss function commonly used for binary classification tasks. It integrates the binary cross-entropy loss with the sigmoid activation function, making it suitable for directly calculating the loss from the logits output of the model. In an unbalanced dataset, where the number of positive and negative samples varies greatly, using standard BCEWithLogitsLoss may result in poor predictions for the minority class. To address this issue, we utilized the pos_weight to amplify the contribution of the minority class in the loss calculation, enhancing the model’s focus on this underrepresented category. The corresponding formula is as follows:


(9) \begin{eqnarray*}
BCEWithLogitsLoss ({x,y}) &=& \frac{1}{N}\mathop \sum \limits_{i = 1}^N \left[-\omega \cdot {y}_{i} \cdot \log (\sigma ({x}_{i}))\right.\nonumber\\ &&\left.- (1 - {y}_{i}) \cdot \log (1 - \sigma ({x}_{i}))\right]\nonumber\\
\end{eqnarray*}

where $N$ denotes the number of samples; $\omega$ represents the pos_weight; ${{x}_i}$ corresponds to the logits output by the model without any activation function processing; ${{y}_i}$ signifies the true label of sample $i$, taking a value of either 0 or 1; and the sigmoid function is defined as $\sigma ( x ) = \frac{1}{{1 + {{e}^{ - x}}}}$, which converts the logits into probabilities. The value of pos_weight is typically calculated based on the ratio of positive and negative samples in the dataset. If the number of positive samples is less than the number of negative samples, pos_weight is set as the ratio of negative to positive samples, and vice versa.

### Evaluation metrics

Accuracy (ACC), Sensitivity (SN), Specificity (SP), and the Matthews Correlation Coefficient (MCC) were selected to evaluate the performance of the model comprehensively [83, 84]. The formulas for these metrics are as follows:


(10) \begin{eqnarray*}
ACC = \frac{{TP + TN}}{{TP + TN + FP + FN}}
\end{eqnarray*}

(11) \begin{eqnarray*}
SN = \frac{{TP}}{{TP + FN}}
\end{eqnarray*}

(12) \begin{eqnarray*}
SP = \frac{{TN}}{{TN + FP}}
\end{eqnarray*}

(13) \begin{eqnarray*}
MCC = \frac{{TP \times TN - FP \times FN}}{{\sqrt {\left( {TP + FP} \right)\left( {TP + FN} \right)\left( {TN + FP} \right)\left( {TN + FN} \right)} }}\nonumber\\
\end{eqnarray*}

Here, TP represents the number of correctly predicted positive samples; TN represents the number of correctly predicted negative samples; FP represents the number of negative samples incorrectly predicted as positive; and FN represents the number of positive samples incorrectly predicted as negative. the receiver operating characteristic (ROC) curve and its area under the curve (AUC) [85], the precision-recall (PR) curve and its area under the precision-recall curve (AUPR) [86], and the confusion matrix [87] were also employed as evaluation metrics.

## Results

### Evaluation results at different classification stages

**Figure 3.**
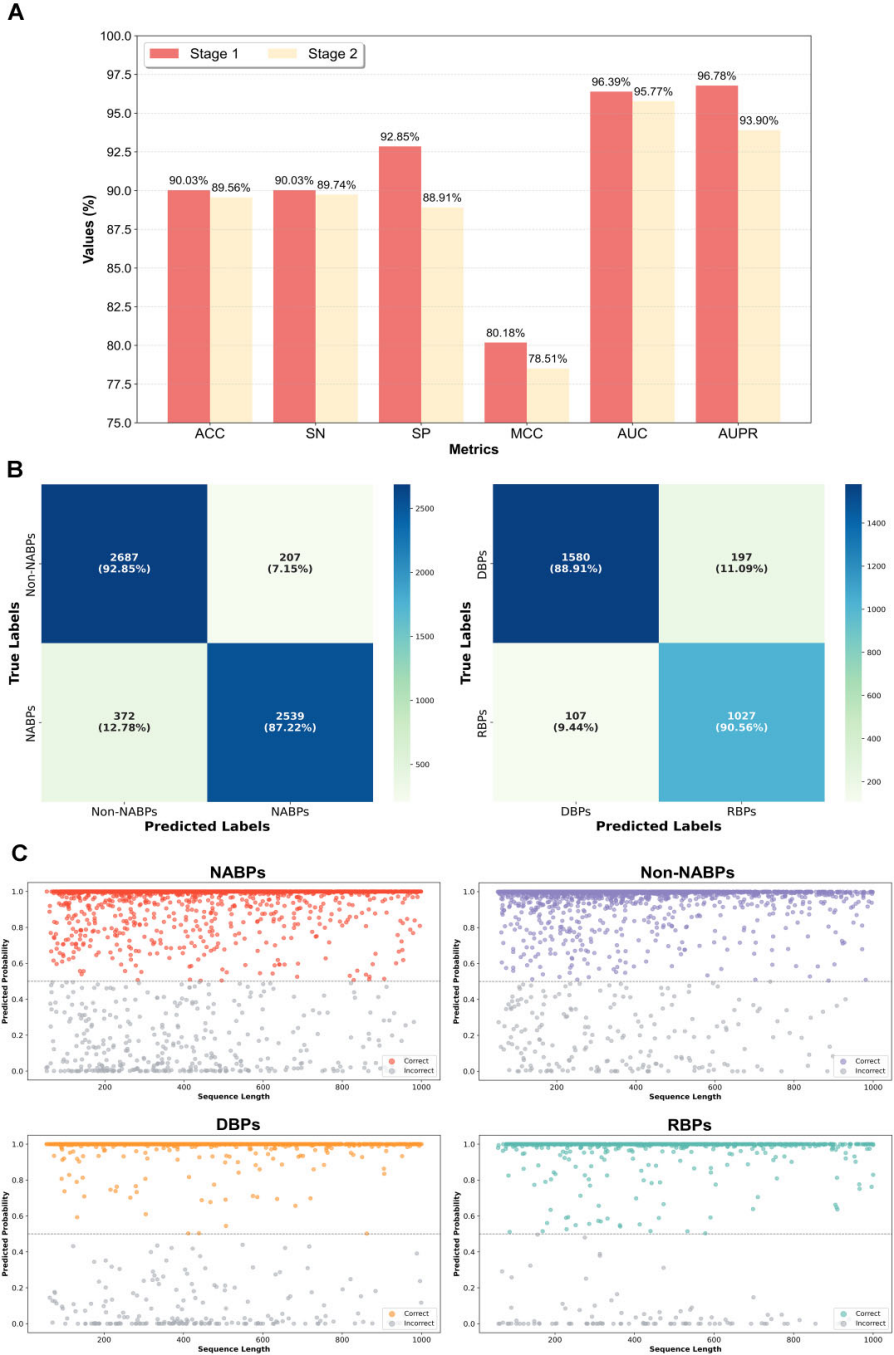
Evaluation results. (**A**) Performance of the model on Test_data at different stages. (**B**) Confusion matrices at different stages. Stage 1: NABPs versus non-NABPs. Stage 2: DBPs versus RBPs. (**C**) Prediction results for each sequence across various protein types. The horizontal axis represents the length of the protein sequences (ranging from 50 to 1000), while the vertical axis shows the predicted probability. A threshold of 0.5 is set on the vertical axis: values above 0.5 signify correct predictions, with values closer to 1 representing a higher probability of accuracy; values below 0.5 denote incorrect predictions, with values closer to 0 indicating a higher probability of error.

The experimental results in this study are reported as the average values from stratified five-fold cross-validation. As shown in Fig. 3A, in the first stage, the model classified NABPs and non-NABPs with an ACC of 90.03% and an MCC of 80.18%; in the second stage, the model categorized DBPs and RBPs with an ACC of 89.56% and an MCC of 78.51%. Additionally, for the first stage, the AUC and AUPR were 96.39% and 96.78%, respectively; for the second stage, the AUC and AUPR were 95.77% and 93.90%, respectively. To provide a comprehensive performance evaluation across multiple folds and reduce potential biases from individual folds, the average confusion matrix was calculated by averaging the confusion matrices of all folds, as illustrated in Fig. 3B. Based on this, Fig. 3C depicted the prediction performance of each protein sequence in the test set in the form of a scatter plot.

### Ablation study

To assess the impact of each component of the model on overall performance, this study designed a series of ablation studies. The specific methods are as follows:

- ESM-2: Features are extracted solely using ESM-2 and directly input into the classifier.
- E2-TC (ESM-2-Transformer-CNN): Based on ESM-2, classification is performed by incorporating Path 1.
- E2-BLA (ESM-2-BiLSTM-Attention): Based on ESM-2, classification is performed by incorporating Path 2.
- DRBP-EDP: This is the complete model, including ESM-2, Path 1 and Path 2. It integrates features extracted from different paths through feature fusion, aiming to leverage the complementary advantages of each module.

**Figure 4.**
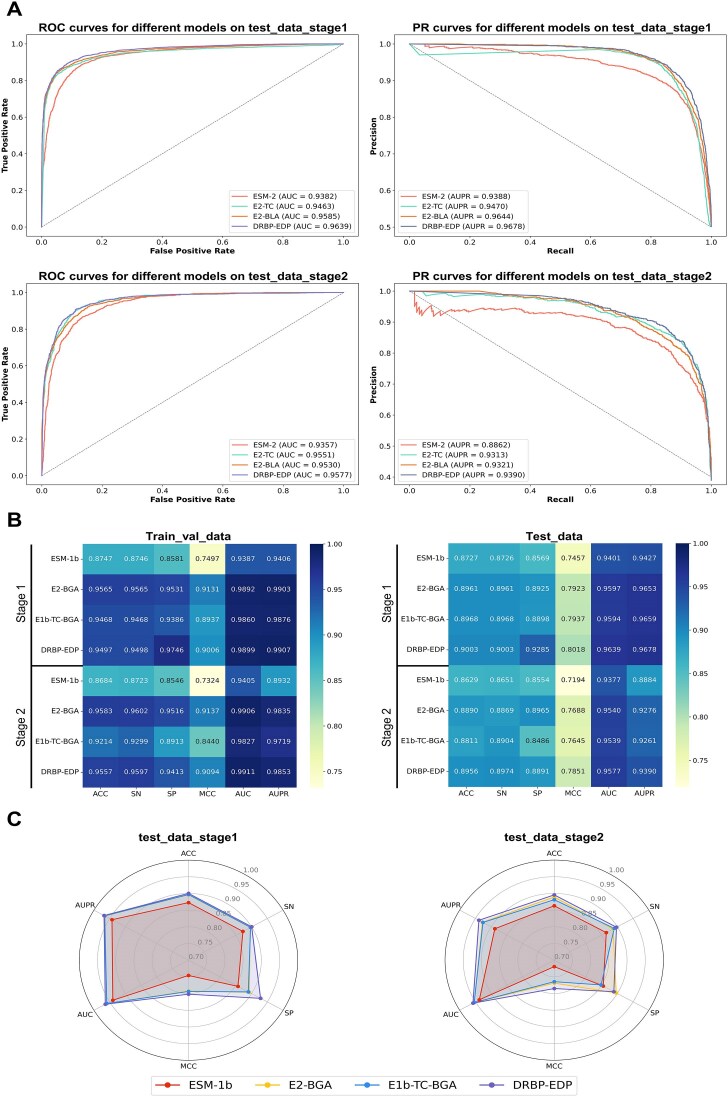
Results of ablation and comparison experiments. (**A**) ROC and PR curves for different models on Test_data. (**B**) Performance of different models on Train_val_data and Test_data (Stage 1 and Stage 2). (**C**) Performance of different models on Test_data.

Each method was trained on Train_val_data to derive the optimal model states in the two stages, which were then evaluated on the independent Test_data. As shown in Table 3, in the first stage, DRBP-EDP achieved the best performance across all metrics, with an improvement of 2.52% in ACC and 5.10% in MCC compared to ESM-2, demonstrating the effectiveness of the dual-path feature extraction and fusion strategy. In the second stage, E2-TC outperformed E2-BLA in ACC by 0.86% and DRBP-EDP in SP by 2.14%. Although E2-TC and E2-BLA, as single-path neural network integration methods, achieved commendable results, their AUC and AUPR were inferior to those of DRBP-EDP. This indicates that the combination of ESM-2 with the dual-path neural network offers certain advantages in handling complex protein classification tasks. Additionally, Fig. 4A visually presents the overall performance of each method, further highlighting the superior performance of DRBP-EDP.

**Table 3.** Performance of various methods on Test_data at different stages

Classification stage	Method	ACC (%)	SN (%)	SP (%)	MCC (%)	AUC (%)	AUPR (%)
Stage 1	ESM-2	87.51	87.50	85.49	75.08	93.82	93.88
	E2-TC	88.99	89.00	90.19	78.01	94.63	94.70
	E2-BLA	89.65	89.65	92.29	79.41	95.85	96.44
	DRBP-EDP	**90.03**	**90.03**	**92.85**	**80.18**	**96.39**	**96.78**
Stage 2	ESM-2	86.53	86.58	86.38	72.25	93.57	88.62
	E2-TC	89.08	88.52	**91.05**	77.03	95.51	93.13
	E2-BLA	88.22	87.92	89.25	75.41	95.30	93.21
	DRBP-EDP	**89.56**	**89.74**	88.91	**78.51**	**95.77**	**93.90**

**Note:** The best results are shown in bold.

### Comparative performance of model architectures on the benchmark dataset NR40

This study aims to evaluate the performance differences among various model architectures in protein classification tasks through comparative experiments. We selected the following four models for comparison:

- ESM-1b [88]: A PLM pre-trained on Uniref50, which, similar to ESM-2 used in this study, comprises approximately 650 M parameters, 33 Transformer layers (referred to as EsmLayers), and an embedding dimension of 1280.
- E2-BGA (ESM-2-BiGRU-Attention): Based on ESM-2, the BiLSTM in Path 2 is replaced by BiGRU [89], which has fewer parameters and lower computational complexity compared to BiLSTM.
- E1b-TC-BGA (ESM-1b-Transformer-CNN-BiGRU-Attention): Based on ESM-1b, the BiLSTM in Path 2 is replaced by BiGRU, and then combined with Path 1 to form a new dual-path structure for classification.
- DRBP-EDP: Based on ESM-2 and combining both Path 1 and Path 2, this serves as the final model for a comprehensive comparison.

At each stage, the models were trained on Train_val_data under identical conditions and subsequently evaluated on Train_val_data and Test_data, respectively. As illustrated in Fig. 4B, all models performed well on Train_val_data, with ACC exceeding 86%, indicating effective fitting to the training data. Moreover, the differences in ACC between Train_val_data and Test_data for each model were all within 7%, suggesting that none of the models exhibited overfitting. Fig. 4C further demonstrates the performance of different models on Test_data, facilitating the comparative analysis among multiple metrics. It can be seen that, except for the SP in the second stage being 0.74% lower than that of E2-BGA, DRBP-EDP achieved the optimal performance in all other metrics.

Through the analysis of Table 3 and Fig. 4B, we provided a detailed comparison of the methods in ablation studies and comparative experiments. The results revealed that in both classification stages, the ACC and MCC of ESM-2 were higher than those of ESM-1b, while the AUC and AUPR were slightly lower, with the differences in these metrics being less than 1%, indicating that the difference in overall performance between ESM-2 and ESM-1b is not significant. Similarly, due to the architectural similarities, the experimental results of E2-BLA and E2-BGA were comparable, suggesting that the difference in effect between BiLSTM and BiGRU is not noticeable. Finally, E1b-TC-BGA containing ESM-1b and BiGRU was compared with DRBP-EDP comprehensively, but the results did not indicate similar performance. DRBP-EDP outperformed E1b-TC-BGA across the board, particularly with SP in the first and second stages being 3.87% and 4.05% higher, respectively, thereby demonstrating the superior effectiveness of combining ESM-2 with a dual-path neural network in protein classification tasks.

### Comparative performance analysis of DRBP-EDP with other predictors

To systematically evaluate the predictive capabilities of DRBP-EDP, we benchmarked it against 13 predictors across diverse NABPs prediction tasks. These include specialized methods for DBPs [10–12], RBPs [16, 17, 44], and multi-object frameworks (DBPs/RBPs) [18, 19, 45, 46, 51, 52]. Performance was evaluated on two critical datasets: the temporal validation set TEST236 and cross-species datasets from five organisms (*A. thaliana, Human, S. cerevisiae, Mouse, C. elegans*). To ensure fair benchmarking against class imbalance and task-specific biases, global metrics (ACC, MCC) were evaluated across all samples, while class-specific SN and SP were macro-averaged [90].

As summarized in Table 4, DRBP-EDP demonstrated unprecedented performance, achieving 93.64% ACC and 90.54% MCC, surpassing the second-ranked predictor (iDRPro-SC) by 5.50% and 7.68%, respectively. Traditional machine learning approaches (e.g. PlDBPred, DPP-PseAAC) exhibited limited discriminative power (MCC < 20%), while hybrid deep learning frameworks like DeepDRBP-2L (MCC = 66.10%) and iDRBP-ECHF (MCC = 72.37%) showed intermediate performance. Notably, DRBP-EDP achieved 96.84% SP, reducing false positives by 2.71% (versus iDRPro-SC’s 94.13% SP) to 40.39% (versus PlDBPred’s 56.45% SP). The method balanced class-wise performance with a minimal SN-SP gap, demonstrating its robustness in distinguishing DBPs, RBPs, and non-NABPs.

**Table 4.** Performance comparison with existing predictors on TEST236

Method	Prediction types	Methods used	ACC (%)	SN (%)	SP (%)	MCC (%)
PlDBPred	DBPs	SVM	61.44	63.25	56.45	7.95
DPP-PseAAC	DBPs	RF + SVM_RFE	61.44	73.27	61.46	18.02
ProkDBP	DBPs	LightGBM + RF_VIM	71.19	82.97	66.57	40.32
Deep-RBPPred (balance)	RBPs	CNN	51.69	62.58	56.04	23.61
Deep-RBPPred (unbalance)	RBPs	CNN	60.17	71.20	60.16	28.02
CatRAPID signature	RBPs	SVM_RBF	65.25	67.34	58.33	28.63
RBPLight	RBPs	LightGBM	80.08	87.64	68.06	57.41
DeepDRBP-2L	DBPs/RBPs	CNN + BiLSTM	77.12	77.48	88.52	66.10
iDRBP-ECHF	DBPs/RBPs	RF + ERT + CNN + LSTM	80.08	80.58	90.18	72.37
iDRBP_MMC	DBPs/RBPs	CNN_motif	80.51	80.97	90.38	72.51
iDRBP-EL	DBPs/RBPs	ERT	83.05	83.41	91.58	75.19
IDRBP-PPCT	DBPs/RBPs	RF	85.17	85.44	92.58	78.30
iDRPro-SC	DBPs/RBPs	BiLSTM + RF	88.14	88.44	94.13	82.86
DRBP-EDP	DBPs/RBPs	ESM-2 + Dual-path NN	**93.64**	**93.78**	**96.84**	**90.54**

**Note:** The best results are shown in bold.

DRBP-EDP demonstrated superior cross-species generalizability compared to iDRBP-EL and iDRPro-SC, achieving the highest MCC across all tested organisms (Table 5), with mean MCC improvements of 20.7% (versus iDRBP-EL) and 11.5% (versus iDRPro-SC). Notably, DRBP-EDP’s SP remained consistently above 93% in all species, including evolutionarily distant organisms [91] (*S. cerevisiae*: 94.51% SP), where conventional methods suffered severe performance decay (e.g. iDRBP-EL’s MCC dropped to 41.87%). Despite iDRPro-SC’s marginally higher SP in *C. elegans* (94.14% versus 93.18%), DRBP-EDP achieved a 3.35% MCC improvement (79.94% versus 76.59%), prioritizing functional accuracy over raw specificity. The method maintained SN-SP gaps below 6% for all species (e.g. *Human*: 89.99% SN versus 95.54% SP), demonstrating reliable performance in real-world applications requiring high-confidence predictions.

**Table 5.** Cross-species performance benchmarking of DRBP-EDP against iDRBP-EL and iDRPro-SC

Dataset	Method	ACC (%)	SN (%)	SP (%)	MCC (%)
	iDRBP-EL	85.61	83.64	92.53	77.51
*A. thaliana*	iDRPro-SC	87.77	86.40	93.89	81.02
	DRBP-EDP	**95.20**	**95.34**	**97.55**	**92.57**
	iDRBP-EL	77.83	78.52	89.46	68.23
*Human*	iDRPro-SC	84.82	82.37	91.62	77.34
	DRBP-EDP	**91.33**	**89.99**	**95.54**	**86.74**
	iDRBP-EL	54.86	66.38	80.54	41.87
*S. cerevisiae*	iDRPro-SC	77.12	80.34	88.72	63.69
	DRBP-EDP	**88.40**	**88.77**	**94.51**	**80.28**
	iDRBP-EL	69.08	75.34	85.58	57.27
*Mouse*	iDRPro-SC	80.68	81.39	89.77	67.58
	DRBP-EDP	**90.34**	**91.27**	**95.81**	**84.11**
	iDRBP-EL	88.18	78.68	91.31	75.22
*C. elegans*	iDRPro-SC	88.18	80.70	**94.14**	76.59
	DRBP-EDP	**90.00**	**87.71**	93.18	**79.94**

**Note:** The best results are shown in bold.

### Impact of sequence redundancy thresholds on overfitting and predictive performance

**Figure 5.**
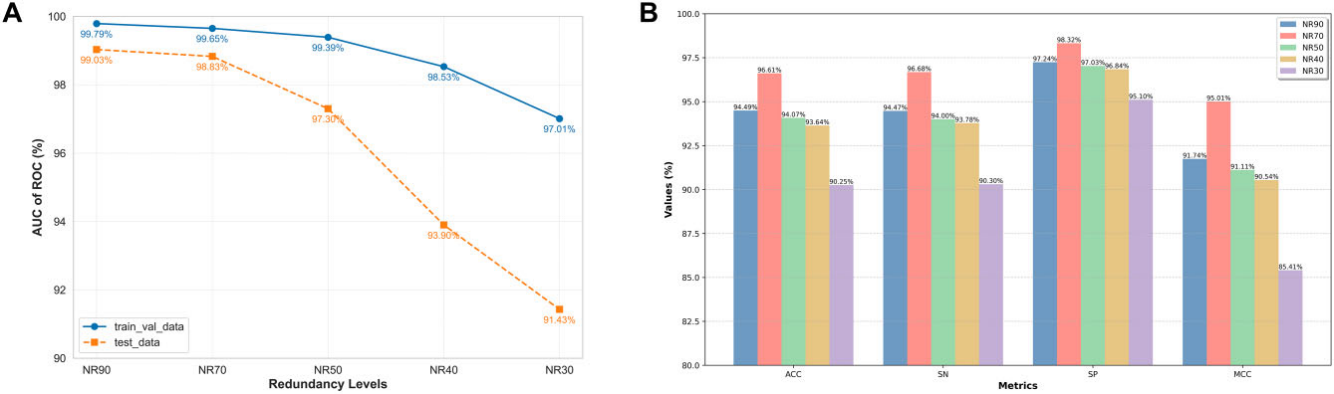
Performance of the model trained on datasets with varied sequence redundancy thresholds. (**A**) Performance evaluation on threshold-partitioned training-validation and test sets. (**B**) Generalization capability on TEST236.

As depicted in Fig. 5A, a pronounced performance gap emerges between the train_val_data and test_data at NR40 (98.53% versus 93.90%). This demonstrates that the threshold not only maintains high accuracy but also effectively mitigates overfitting, thereby revealing a critical connection between sequence redundancy thresholds and overfitting dynamics during training. Mechanistically, lower data redundancy may restrict the model’s ability to adequately learn complex sequence features due to insufficient diversity in training samples. Conversely, while increased redundancy enhances training precision, the stagnant test accuracy suggests that the model begins memorizing noise or dataset-specific idiosyncrasies in the training data, ultimately degrading generalization performance.

In Fig. 5B, peak performance (MCC = 95.01%) is attained at NR70, surpassing both lower-redundancy (NR50: MCC = 91.11%) and higher-redundancy (NR90: MCC = 91.74%). These results demonstrate that ESM-2 effectively captures critical information embedded in sequences, while simultaneously revealing that performance does not monotonically improve with increasing sequence quantity. The existence of an optimal threshold (NR70) indicates a critical balance between sequence redundancy and model generalizability, where the synergy of these factors maximizes predictive capability. This non-linear relationship further supports the hypothesis that appropriate redundancy management enables optimal feature learning while avoiding data-driven over-specialization.

### Application

**Figure 6.**
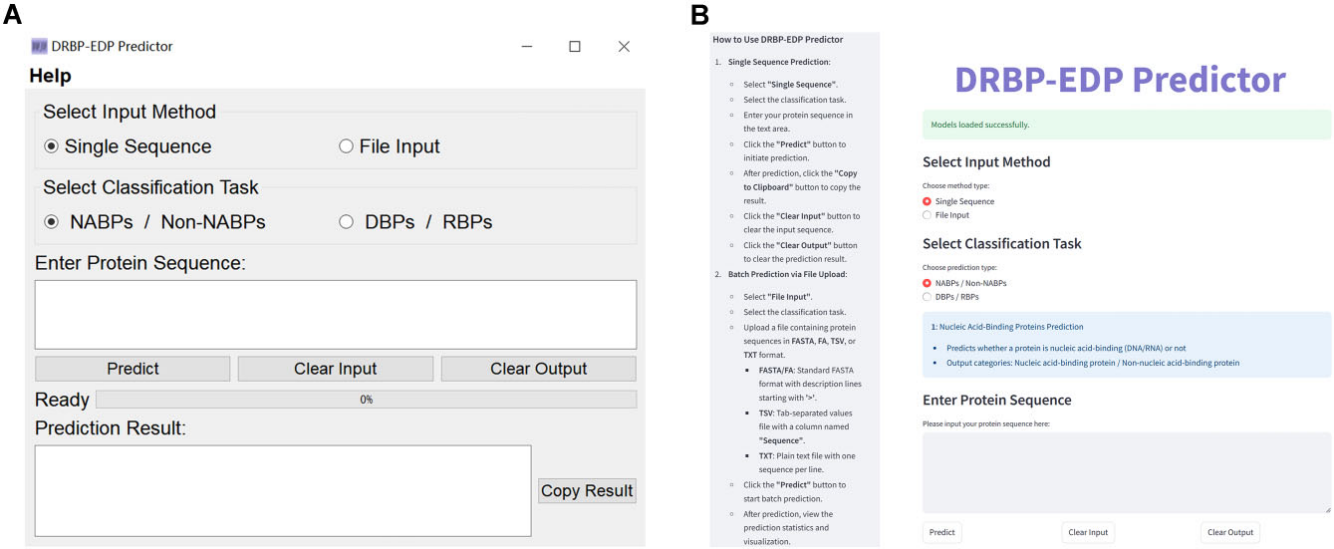
Interface of DRBP-EDP. (**A**) The executable version. (**B**) The web-based version.

To enhance accessibility and usability, DRBP-EDP has been developed in both executable and web-based versions. The executable version (Fig. 6A) allows users to run the application locally, ensuring faster performance for large-scale classification, while the web-based version (Fig. 6B) offers the convenience of cloud-based computation, accessible through any modern browser without the need for installation.

## Discussion

Previous studies have demonstrated various characteristics and limitations in the selection of datasets, construction of models, and application of classification methods. To gain a deeper understanding of the progress and existing problems in the current field, this paper discusses the following three aspects:

- Datasets. In this study, we have created high-quality protein sequence datasets dedicated to the classification of DBPs and RBPs and have designed a refined dataset construction method for subsequent studies. Many benchmark datasets used in existing literature have become outdated due to scientific advancements and the passage of time. With the discovery of new protein sequences and improved annotations, these datasets have not been updated in a timely manner, resulting in their failure to encompass the latest scientific findings. Moreover, some datasets suffer from the issue of data leakage, where identical or highly similar protein sequences are present in both the training and testing sets. This phenomenon can lead to inflated model evaluation results, failing to accurately reflect the actual generalization capability of the model. Therefore, to obtain more reliable and representative model performance evaluations, it is essential to construct new benchmark datasets that ensure the recency, completeness, and independence of the data. In our benchmark dataset, the ratio of NABPs to non-NABPs is approximately 1:1, while the ratio of DBPs to RBPs is around 3:2. We believe that as protein databases continue to expand, the quality of the dataset will improve, and the proportions of different protein classes will gradually reach equilibrium.
- Model architecture. This study proposes a model architecture that integrates the PLM ESM-2 with a dual-path neural network, aiming to overcome the limitations of traditional methods. Existing research predominantly utilizes conventional feature extraction techniques or feature encoding approaches to process protein sequences, followed by the application of a single-path neural network for protein classification tasks. First, these methods rely on predefined rules for feature extraction, which easily leads to information loss and makes it difficult to capture the global and complex patterns within protein sequences. Second, they tend to overlook the contextual dependencies between amino acids in the sequence, thereby underutilizing the interactions at different positions in the sequence. Additionally, single-path neural networks are insufficient in extracting higher-order features and managing long-range dependencies, limiting the feature representation and prediction accuracy of the model. Lastly, the subjectivity in feature selection and the dependence on specific datasets restrict the generalization capability of the model, making it challenging to achieve stable performance on different datasets or new samples.
  In contrast, the PLM can automatically learn high-dimensional and complex features from sequences, avoiding the tedious work of manual feature design and potential subjective biases. The dual-path neural network effectively enhances the expressive power and predictive performance of the model by processing different feature channels in parallel and fusing multi-scale information. In this study, the implementation of a reasonable data partitioning strategy and an optimized loss function successfully improved the classification performance of the model on both balanced and unbalanced datasets. Nevertheless, there remains room for improvement in the SP of the model in the second classification stage. Due to hardware constraints, we were unable to utilize ESM2 with larger parameters, which might have limited the depth and nuance of feature representations. Future research should explore multi-path neural networks, increasing the number of pathways to capture a broader diversity of features. Recent advances in PLMs, exemplified by SaProt [92], DPLM-2 [93], and ESM3 [94], have demonstrated remarkable capabilities in learning biologically meaningful representations from protein sequences and structural features through self-supervised pretraining. These models effectively capture hierarchical patterns associated with protein functions, such as subcellular localization [20, 95], differential binding preferences between single-stranded DNA and double-stranded DNA [96], and nucleic acid-binding site prediction [31, 32]. We hypothesize that the structure-aware vocabulary in SaProt could be adapted for the prediction of NABPs, as this method integrates both protein sequence and structural information, enabling the identification of binding specificity determinants that remain elusive in sequence-only analyses. However, empirical validation of this hypothesis remains to be conducted.
- Classification method. In this study, we propose a phased classification approach, in which protein sequences are firstly classified as NABPs or non-NABPs in the first stage, and then NABPs are further subdivided into DBPs or RBPs in the second stage. Compared with existing binary classification methods, such as directly distinguishing between DBPs and non-DBPs, RBPs and non-RBPs, or DBPs and RBPs, this method reduces ambiguity through a stepwise filtering process to enhance classification accuracy and simplifies the complexity of the problem to render the objectives of each phase more explicit. However, a limitation of this method is its inability to effectively analyze proteins with both DNA- and RNA-binding functions, as it only classifies NABPs as DBPs or RBPs without considering the multi-binding capability. Therefore, it is recommended to introduce multi-label classification mechanisms and other solutions in subsequent studies to more accurately characterize the multifunctionality of proteins, thereby enhancing a comprehensive understanding of their functions.

## Conclusion

In this work, we presented DRBP-EDP, a method for classifying DBPs and RBPs by integrating a PLM with a dual-path neural network. The method leverages ESM-2 with powerful feature extraction capabilities and combines a dual-path neural network containing Transformer, CNN, BiLSTM, and Attention to achieve phased protein classification. Specifically, in the first stage, protein sequences are classified into NABPs or non-NABPs; in the second stage, the NABPs are further categorized into DBPs or RBPs. To ensure the reliability of model training and evaluation, we designed a refined dataset construction approach and created high-quality protein classification datasets. The results demonstrated that DRBP-EDP outperformed other methods in terms of classification performance. Future research should explore more diverse network architectures and multi-label classification mechanisms to further uncover the latent information in protein sequences, enhance the generalization ability of the model, and deepen the understanding of multifunctional proteins. Our study not only offers novel insights and methods for protein classification but also provides a new tool for protein function research, creating new opportunities for advancements in life sciences.

## Data Availability

The dataset and the code used in this study, along with the application, are available at https://doi.org/10.5281/zenodo.14092184 and https://github.com/MuQiang-MQ/DRBP-EDP.
